# Antimicrobial Resistance Profiles and Genetic Typing of *Salmonella* Serovars from Chicken Embryos in China

**DOI:** 10.3390/antibiotics10101156

**Published:** 2021-09-24

**Authors:** Yaohui Xu, Xiao Zhou, Zenghai Jiang, Yaru Qi, Abdelaziz Ed-Dra, Min Yue

**Affiliations:** 1 College of Veterinary Medicine, Henan University of Animal Husbandry and Economy, Zhengzhou 450046, China; 80588@hnuahe.edu.cn (Y.X.); 80147@hnuahe.edu.cn (Z.J.); q15837181937@163.com (Y.Q.); 2 Department of Veterinary Medicine, Institute of Preventive Veterinary Sciences, College of Animal Sciences, Zhejiang University, Hangzhou 310058, China; xiaozhou@zju.edu.cn; 3 Hainan Institute of Zhejiang University, Sanya 572025, China; abdelaziz_iaa@yahoo.fr; 4 National Clinical Research Center for Infectious Diseases, National Medical Center for Infectious Diseases, State Key Laboratory for Diagnosis and Treatment of Infectious Diseases, The First Affiliated Hospital, College of Medicine, Zhejiang University, Hangzhou 310003, China; 5 Zhejiang Provincial Key Laboratory of Preventive Veterinary Medicine, Hangzhou 310058, China

**Keywords:** *Salmonella*, antimicrobial resistance, antimicrobial resistance genes, PFGE, ESBLs, chicken embryos

## Abstract

*Salmonella* continues to be a major food and public health burden worldwide that can threaten human health via eating contaminated meats, particularly those originating from chicken. In this study, the antimicrobial resistance profiles, epidemiological characteristics of resistance genes, and pulsed field gel electrophoresis (PFGE-XbaI) typing of 120 non-Pullorum/Gallinarum *Salmonella* isolates recovered from chicken embryos in Henan province were determined. The antimicrobial resistant phenotypes and evaluation of the extended-spectrum beta-lactamases (ESBLs) producing strains of *Salmonella* were investigated by the Kirby–Bauer test and the double-disk synergy test. Additionally, 37 antimicrobial resistance genes encoding resistance to five different categories, including aminoglycosides, cephalosporins, sulphonamides, tetracyclines, and β-lactams, were examined by conventional PCR. However, genotyping analysis was conducted by macro-restriction using enzyme XbaI followed by the separation of the restricted DNA fragments by PFGE. The results of this study showed that the studied *Salmonella* strains were highly resistant to ampicillin (66.67%) and sulfisoxazole (66.67%), while they were all susceptible to meropenem, imipenem, colistin, and chloramphenicol. Additionally, 67.5% (81/120) of the studied strains were multidrug resistant, and 21.67% (26/120) were phenotypically confirmed as ESBLs positive. The statistical analysis showed that resistance depends on the serovars, and ESBLs positive strains showed more multi-resistance than ESBLs negative strains (*p* < 0.05). The genotypic antimicrobial resistance showed the detection of 14 among the 37 tested genes, and the concordance between genotypic and phenotypic antimicrobial resistance ranged from 0% to 100% depending on the serovars. However, the PFGE-XbaI typing results showed that the examined *Salmonella* strains were divided into 22 individual subtypes and were grouped in nine clusters, with similarity values ranging from 64.7% to 100%. From this study, we can conclude that the antimicrobial resistance of *Salmonella* serovars isolated from chicken embryos in Henan province was alarming, with rigorous multidrug resistance, which requires the urgent mitigation of the use of antimicrobial drugs in chicken hatcheries. Additionally, our results showed evidence of the presence of different PFGE patterns among the studied *Salmonella* serovars, suggesting the presence of different sources of contamination.

## 1. Introduction

The World Health Organization (WHO) estimates that 600 million people fall ill and that 420,000 people die each year after consuming contaminated food, while USD 110 billion is lost each year in productivity and medical expenses resulting from unsafe food in low- and middle-income countries [1]. Foodborne diseases hamper socioeconomic development via stressing health care systems and harm national economies, tourism, and trade [1]. In this regard, *Salmonella* is classified at the top of the list of major foodborne pathogens, affecting millions of people every year, and is of great importance to food and public health worldwide. In China, 70% to 80% of bacterial food poisonings were linked to *Salmonella* [2]. In fact, this bacterium can directly threaten human health after eating contaminated foods, especially poultry meat, which appears to be one of the major sources of human infection [3,4,5]. Recently, several studies have evaluated the prevalence of *Salmonella* in poultry products [4,5,6,7,8].

The overuse of antimicrobial drugs in agriculture for a long time, especially in animal husbandry, has led to the increase of the antimicrobial resistance of pathogenic bacteria. Under the selection pressure of antibiotic use, bacteria develop antimicrobial genetic determinants that are responsible for bacterial resistance [9,10]. Then, these antimicrobial genetic determinants are horizontally transmitted to other critical pathogenic bacteria via different mechanisms [9,11]. In recent years, the antimicrobial resistance of bacteria was considered among the most major threats to public health worldwide [10,12,13,14]. Regarding *Salmonella* isolates, recent studies have demonstrated their resistance to many critical antimicrobial drugs, including polymyxin, β-lactams, and fluoroquinolones [15,16,17,18,19,20,21,22]. Indeed, these antimicrobial resistant strains can eventually be transmitted to humans through the food chain, causing severe infectious diseases [13,23,24,25,26,27].

The molecular typing of foodborne pathogens is of great importance in epidemiological investigations, allowing the genetic discrimination of isolates and determining the causal agent when an outbreak occurs. Pulsed-field gel electrophoresis (PFGE) is considered among the major standard typing methods for foodborne pathogens and has good repeatability and high discrimination and has been widely applied in typing *Salmonella* isolates recovered from different samples [28,29,30]. However, we noted a lack in the genotypic discrimination of *Salmonella* isolates from chicken embryos in breeder hatcheries.

China is considered a major chicken consuming country, and Henan is the major breeding province. Additionally, chicken and its products are considered important vehicles for the transmission of antimicrobial resistant *Salmonella*. Therefore, this study aims to investigate the antimicrobial resistance of *Salmonella* serovars from the chicken embryos of breeder hatcheries in different regions of Henan province, China, and to trace the genetic relationship of *Salmonella* in different regions and, in particular, among different farms.

## 2. Results

### 2.1. Antimicrobial Resistance and Multidrug Resistance Patterns

**Figure 1:**
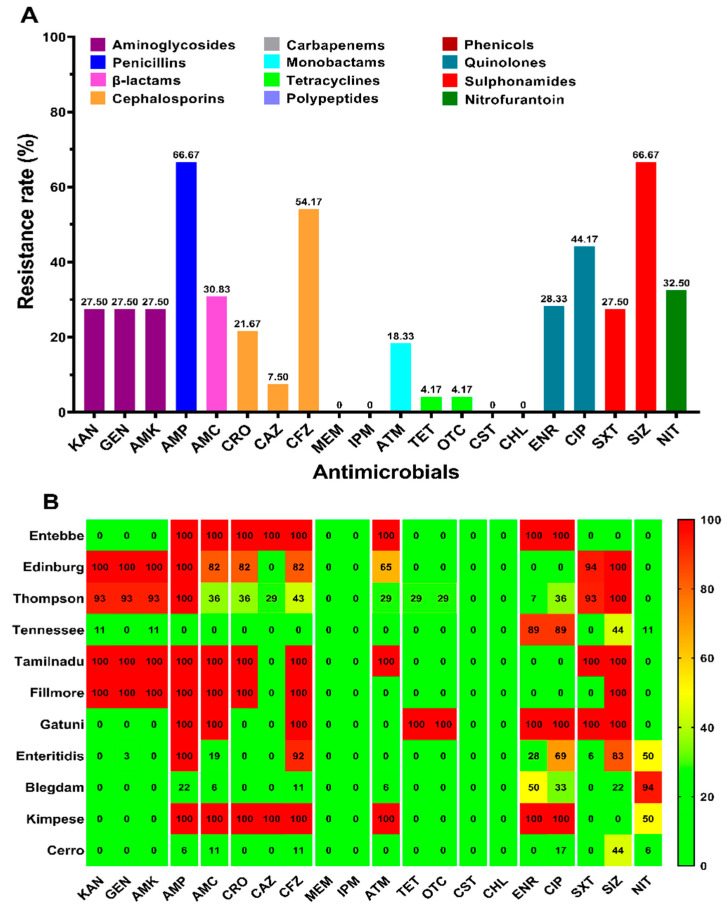
Prevalence of antimicrobial resistance among *Salmonella* isolates. The names of the antimicrobials are abbreviated as kanamycin (KAN), gentamicin (GEN), amikacin (AMK), ampicillin (AMP), amoxicillin-clavulanic acid (AMC), ceftriaxone (CRO), ceftazidime (CAZ), cefazolin (CFZ), meropenem (MEM), imipenem (IPM), aztreonam (ATM), tetracycline (TET), oxytetracycline (OTC), colistin (CST), chloramphenicol (CHL), enrofloxacin (ENR), ciprofloxacin (CIP), trimethoprim-sulfamethoxazole (SXT), sulfisoxazole (SIZ), and nitrofurantoin (NIT). (**A**) The prevalence of antimicrobial resistance among the 120 *Salmonella* isolates against 20 antimicrobial agents of 12 categories. The same category of antimicrobial agents is represented by the same color; (**B**) the distribution of the average antimicrobial resistance (in percent) of various serovars towards 20 antimicrobial agents of 12categories. The color of individual cells varies with the percentage of antimicrobial resistance shown in the cells.

The antimicrobial susceptibility test was conducted for 20 antimicrobial agents of 12 different categories towards 120 *Salmonella enterica* isolates, and the results are shown in Figure 1A. Our results showed that the studied isolates present high resistance to penicillins (ampicillin, 66.67%) and sulphonamides (sulfisoxazole, 66.67%) and quite a high resistance to cephalosporins (cefazolin, 54.17%) and quinolones (ciprofloxacin, 44.17%), while they were all sensitive to carbapenems (meropenem, imipenem), polypeptides (colistin), and phenicols (chloramphenicol). However, the distribution of antimicrobial resistance among the eleven serovars, including Entebbe, Edinburg, Thompson, Tennessee, Tamilnadu, Fillmore, Gatuni, Enteritidis, Blegdam, Kimpese, and Cerro, revealed that the differences in the resistance among the serovars was statistically significant (*p* < 0.05) (Figure 1B).

**Figure 2:**
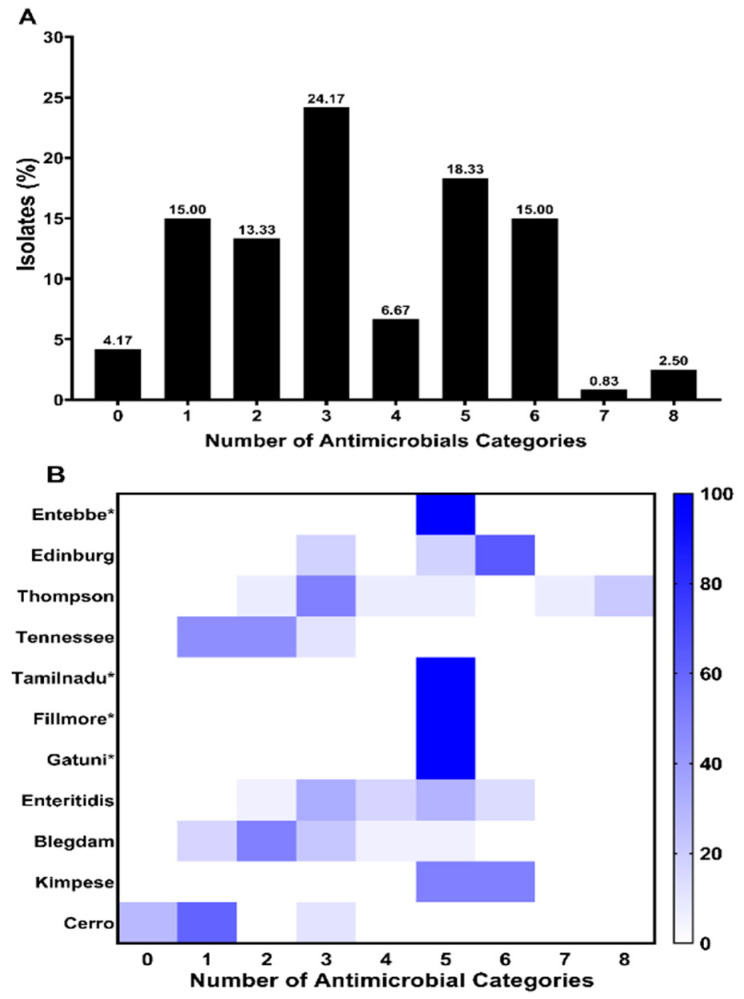
Distribution of multidrug resistance (MDR) strains. (**A**) Prevalence of antimicrobial-resistant strains according to the number of antimicrobial classes; (**B**) serovar distribution of MDR prevalence. The X-axis represents the number of antimicrobial categories. The color of individual cells varies with the percentage of antimicrobial resistance shown in the cells. * serovar containing only one strain.

Multidrug resistance (MDR) is defined as the resistance of isolates to three or more than three antimicrobial classes. Our results showed that 81/120 (67.5%) strains were multidrug resistant (Figure 2A). Additionally, the distribution of MDR among serovars was presented in Figure 2B, where the high MDR rate was observed in *S.* Edinburg (100%, *n* = 17), *S.* Kimpese (100%, *n* = 4), *S.* Enteritidis (94.44%, *n* = 34), *S*. Thompson (92.86%, *n* = 13), and *S.* Blegdam (33.33%, *n* = 6). It is noted that only one strain was identified in the serovars Entebbe, Tamilnadu, Fillmore, and Gatuni, which all presented resistance to five antimicrobial classes (Figure 2B).

### 2.2. Phenotypic ESBL Screening

**Figure 3:**
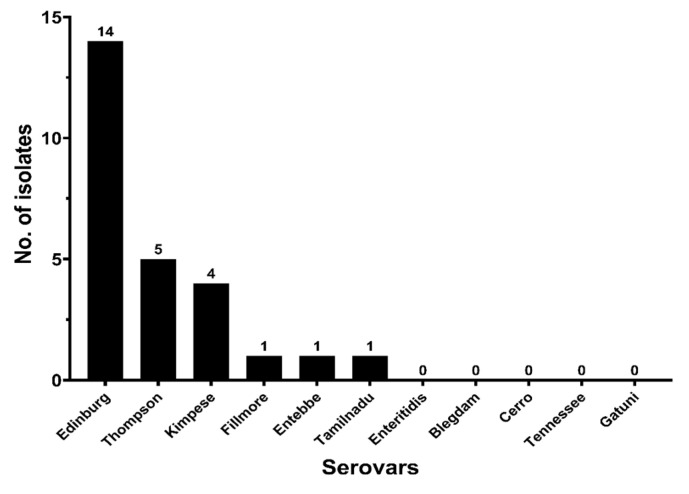
The distribution of ESBLs isolates among the studied serovars.

The double-disk synergy test method was used to phenotypically screen and confirm the ESBLs-producing strains. The results showed that 26 of 120 (21.67%) strains were ESBLs positive (ESBLs+). The distribution of the ESBLs-producing strains among the studied serovars is shown in Figure 3, which reveals that the difference in the ESBLs produced among the serovars was statistically significant (*p* < 0.05). This finding is only valid for the serovars presenting more than four isolates.

### 2.3. Phenotypic-Genotypic Concordance of Drug Resistance

**Figure 4:**
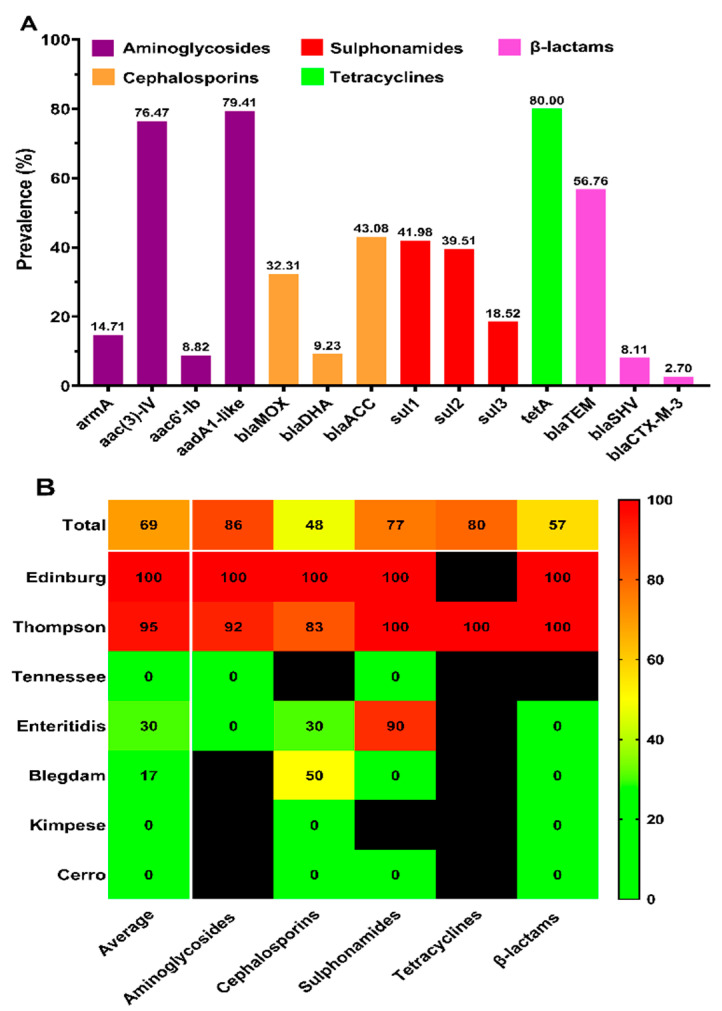
Phenotype-genotype concordance of antimicrobial resistance. (**A**) Prevalence of genotypic drug-resistance among *Salmonella* isolates possessing corresponding phenotypic drug-resistance. Resistant genes belonging to the same class of resistant phenotypes are shown in the same color; (**B**) serovar distribution of concordance between genotypic and phenotypic drug resistance. Each column represents a resistance category, except the first column, which averages the concordance between the genotype and phenotype of the five categories. Due to the small sample size of *S.* Entebbe, *S.* Tamilnadu, *S.* Fillmore, and *S.* Gatuni, each serovar had only one strain, so we only analyzed the remaining seven serovars with relatively large sample sizes. The color of the individual cells varies with the percentage of phenotype–genotype concordance shown in the cells. Cells represented by black mean that the corresponding serovar was phenotypically susceptible and was not tested for the presence of the corresponding resistance genes; thus, the concordance percentage was not calculated.

The detection of antimicrobial resistance genes was conducted by conventional PCR, and the results are presented in Figure 4A. Our results show the detection of 14 antimicrobial-resistant genes among the 37 tested antimicrobial-resistant genes. These genes are distributed as follows: Among the ten tested genes encoding resistance to aminoglycosides, only 4 genes (*armA*, *aac(3)-IV*, *aac6′-Ib* and *aadA1-like*) were detected in 35 aminoglycosides resistant strains, and among the 6 tested genes encoding resistance to cephalosporins, only 3 genes (*bla*_MOX_, *bla*_DHA_ and *bla*_ACC_) were detected in the 65 cephalosporins resistant strains, while among the 12 tested β-lactamase-encoding genes, only 3 genes (*bla*_TEM_, *bla*_SHV_ and *bla*_CTX-M-3_) were detected in the 37 strains resistant to amoxicillin/clavulanic acid (β-lactams combinations). Regarding resistance to sulphonamides, all of the tested genes (*sul1*, *sul2* and *sul3*) were detected in the 81 sulphonamides resistant strains. However, only one (*tetA*) of the six genes encoding resistance to tetracyclines was detected in the five tetracyclines resistant strains.

The drug resistance phenotype–genotype concordance patterns related to different serovars are shown in Figure 4B. Overall, the concordance of the resistance genes and the resistance phenotypes was 69.34%. The detection of the drug-resistant genes corresponding to each drug-resistant phenotype was different, and the phenotype–genotype concordance was uneven in different serovars. In serovars with a relatively large number of phenotypic drug-resistant strains, the corresponding resistance genes were detected approximately in all of the phenotypic resistance strains; for example, the phenotype–genotype drug resistance concordance of the *S.* Edinburg strains was 100%. However, only one phenotypic drug-resistant strain of *S.* Thompson failed to detect the drug resistance gene with a high concordance (95.13%), while the detection rate of drug resistance genes in *S*. Kimpese, *S.* Tennessee, and *S.* Cerro was 0. The remaining serovars, including *S*. Fillmore, *S*. Entebbe, *S*. Gatuni, and *S*. Tamilnadu, which only presented one isolate, were not included in the phenotype–genotype concordance analysis.

### 2.4. PFGE Patterns

**Figure 5:**
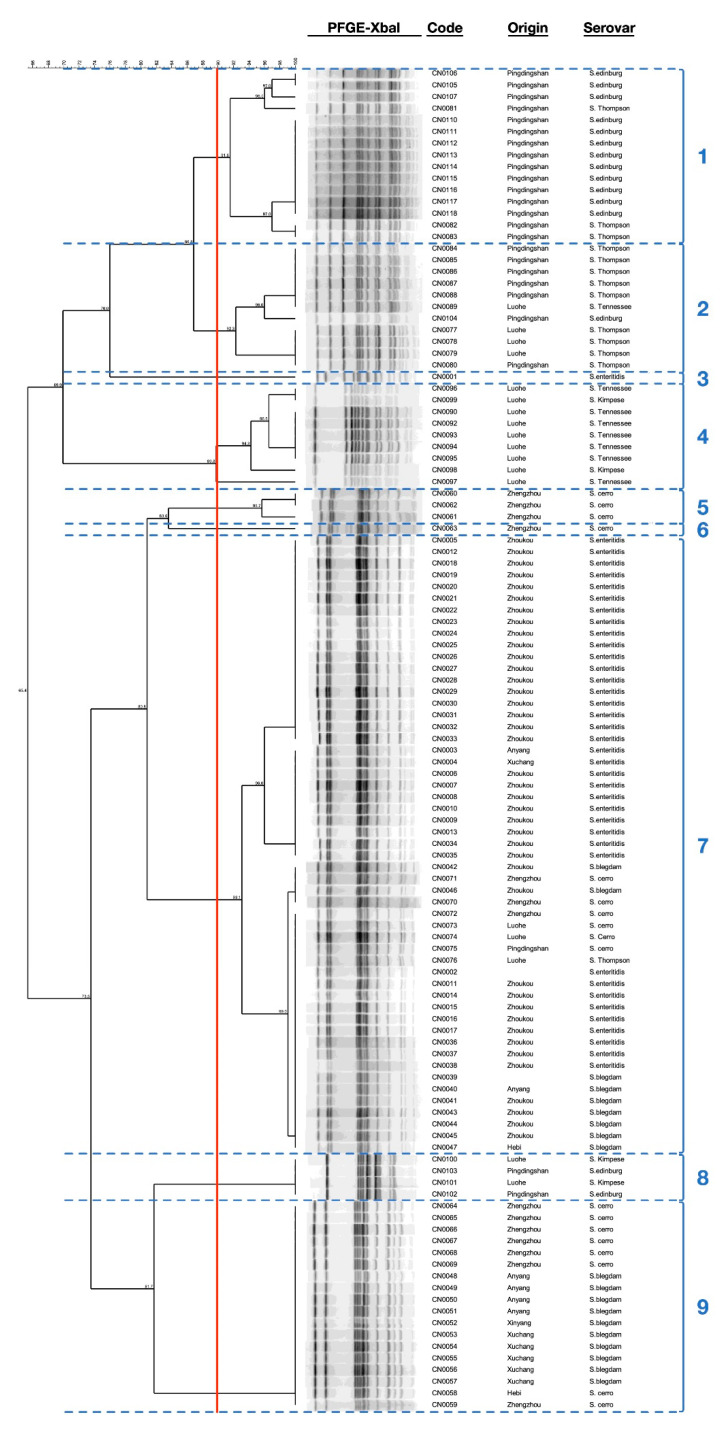
Dendrogram of PFGE profiles of 118 *Salmonella* strains. The analyzed strains were isolated from 28 hatcheries in 9 cities of Henan province, including 115 of 120 isolates, and three strains, including one *S.* Blegdam and two *S.* Enteritidis, were added for expanding analysis.

PFGE XbaI analysis was performed for 118 strains, including 115 of 120 strains, and 3 strains preserved in laboratory (one of *S.* Blegdam and two of *S.* Enteritidis) to expand analysis. The result shows that these strains were divided into 22 different subtypes with similarity values ranging from 64.7% to 100% and were grouped into 9 clusters, and each subtype contained 1 to 21 strains (Figure 5). Among the *S*. Enteritidis isolates analyzed, it was possible to identify four different subtypes, in which one isolate was allocated separately as cluster no. 3 and the other isolates were grouped in cluster no. 7, with similarity values varying between 93.1% to 100%, whereas among the analyzed *S*. Blegdam isolates, three different subtypes were identified, and the isolates were grouped in two different clusters, including no. 7 and 9, with similarity values varying between 73.1% and 100%. Additionally, our results showed that the analyzed *S*. Cerro isolates had high diversity, which were genotypically divided into six different subtypes and that were grouped in four clusters, including no. 5, 6, 7, and 9, with similarity values ranging from 73.5% to 100%. Similarly, we reported high diversity among the *S*. Edinburg isolates with the identification of five different subtypes, in which two isolates were grouped in cluster no. 8, with similarity values of 100%, and the other isolates were grouped in clusters no. 1 and 2, with similarity values ranging between 86.8% and 100%. Moreover, the analyzed *S*. Thompson isolates were also divided into five different subtypes, in which one isolate was placed in cluster no. 7, and the other isolates were grouped in clusters no. 1 and 2, with similarity values varying between 86.8% and 100%. However, among the analyzed *S*. Tennessee isolates, we identified four different subtypes, in which one isolate was placed in cluster no. 4, and the other isolates were grouped together in cluster no. 2, with similarity values ranging between 90% and 100%. Finally, PFGE analysis identified three different subtypes among the analyzed *S.* Kimpese isolates, in which two isolates were grouped in cluster no. 4, with similarity values ranging between 94.3% and 100%, and the two other isolates were grouped in cluster no. 8, with a similarity value of 100%.

## 3. Discussion

*Salmonella* infection remains a major health concern that is common in poultry and is transmitted to humans through the food chain [31,32,33]. It seriously endangers the health of humans and poultry as well as the development of the poultry industry and the economy [34]. Therefore, it is urgent to strengthen non-therapeutic medication management in the poultry industry with guidance and restrictions on prophylactic drug use to reduce the use of antimicrobials in the breeding process so as to alleviate the emergence of drug-resistant and multidrug-resistant strains, thereby reducing the risk of drug-resistant strain transmission to humans through the food chain [16,31,35,36].

All of the 120 strains tested in this study were susceptible to colistin, meropenem, imipenem, and chloramphenicol. It is interesting to note that the use of these antimicrobials is banned by veterinary medicine. While the resistance to nitrofurantoin, which was listed as a banned veterinary drug, was 32.50%. Additionally, the results of this study showed that the antimicrobial resistant strains were mainly obtained from two hatcheries in Zhoukou region, which indirectly indicates the necessity of improving the drug regimen in this area. Moreover, the highest resistance was observed against ampicillin and sulfisoxazole (66.67% for each one), which was consistent with the results reported in other Chinese regions, including Shanghai (AMP: 50.7%; SIZ: 49.32%) [30], Sichuan (AMP: 87.8%) [37], Shandong (AMP: 97.7%) [38], and Guangdong (AMP: 31.8%; SIZ: 70.2%) [39], indicating that the *Salmonella* recovered from chickens had developed serious resistance to ampicillin and sulphonamides; therefore, the use of these drugs should be suspended. However, our results showed moderate resistance to amoxicillin-clavulanic acid (37/120; 30.83%), which was consistent with the results obtained in retail raw poultry meat in China after merging intermediate with resistant results (28%) and higher than those reported in broiler chickens along the slaughtering process in China (8.5%) [37]. On the other hand, we found that the resistance rate to first-generation cephalosporins (CFZ: 54.17%) was much higher than that of third-generation cephalosporins (CRO: 21.67%; CAZ: 7.50%), which was in line with related reports [40]. Importantly, our study showed a high rate of multidrug-resistant strains (67.5%), which was higher than that found in slaughterhouses in Henan (39.8%) [41], and in poultry, swine, and cattle farms in central China, including Henan (34.72%) [42], but was in line with many other studies [8,43,44,45]. The antimicrobial resistance of the studied *Salmonella* strains varies according to serovars, in which the serovars Edinburg and Thompson present the highest resistance. Additionally, this resistance may depend also on the region of isolation and drug regimen.

ESBLs are enzymes that are mainly produced by bacteria belonging to the *Enterobacteriaceae* family under the selective pressure of the use of broad-spectrum β-lactam antimicrobials, especially third-generation cephalosporins. ESBLs have a wide range of enzyme activities and can hydrolyze penicillin, first- or second-generation cephalosporins, and broad-spectrum cephalosporins containing oxime groups, including third-generation cephalosporins such as ceftazidime, cefotaxime, ceftriaxone, and cefixime as well as fourth-generation cephalosporin-like cefepime, and can also hydrolyze monobactams such as aztreonam [46]. Recent studies around the world indicate that the prevalence of ESBLs-producing strains is increasing each year [5,47,48,49]. In our study, the prevalence of ESBLs-producing strains was 21.67%, which was lower than that found in retail chicken in Henan province in 2017 (50.0%) [5]. However, the distribution of ESBLs producing strains according to serovars was as follows: Edinburg (82.35%; 14/17), Thompson (35.71%; 5/14), Blegdam (0%; 0/18), Cerro (0%; 0/18), Tennessee (0%; 0/9), and Enteritidis (0%; 0/36), which was consistent with other reports [5]. It is noted that the serovars presented a number of isolates that were lower than or equal to five were not taken for this comparison and discussion. Additionally, our results showed the resistance of ESBLs-producing strains to other non-β-lactam antimicrobial categories, with high resistance not only to cephalosporins and penicillins but also to aminoglycosides and sulphonamides, which was consistent with other studies demonstrating that ESBLs+ strains are often multidrug resistant [48]. Importantly, the antimicrobial resistance of ESBLs+ bacteria is significantly more serious than that of ESBLs- bacteria. Therefore, it is necessary to combine the antimicrobial susceptibility test with the ESBLs detection test to choose effective antimicrobial agents for prevention and control when dealing with salmonellosis in the clinic. At the same time, taking some measures such as the adoption of antimicrobial synergists or enzyme inhibitors, synergy, and medication rotation can effectively reduce the development of bacterial resistance.

The detection of antimicrobial resistance genes showed that among the targeted genes encoding resistance to aminoglycosides, the prevalence of *aadA1-like* (79.41%) and *aac(3)-IV* (76.47%) was the highest, which was higher than that found previously for *aac(3)-IV* (53.2%) in *Salmonella* recovered from broilers in Sichuan province [37]. However, among the ESBLs+ strains, the dominant resistance gene was *bla*_TEM_ (80.77%), which was in agreement with the results obtained previously in *Salmonella* recovered from retail raw chicken carcasses [5]. Additionally, the detection of genes encoding resistance to tetracyclines showed the presence of only *tetA,* with a prevalence rate of 80%; this result is in accordance with that of previous studies showing the dominance of *tetA* (100%) and *tetB* (67.7%) genes among the tetracycline-resistant *Salmonella* isolates [30]. However, all of the targeted genes encoding resistance to sulphonamides were detected with different proportions (*sul1*: 41.98%, *sul2*: 39.51%, and *sul3*: 18.52%), these results were lower than those obtained from the *Salmonella* isolates recovered from slaughterhouses in Sichuan and from retail chicken in Shanghai [30,37]. According to the statistical analysis, the phenotypic and genotypic antimicrobial resistance of *Salmonella* strains varied greatly in each serovar. In this study, the results of phenotypic resistance of *S.* Edinburg and *S.* Thompson, especially those presenting high-multidrug resistance, were consistent with genotypic profiles (100% and 95.13%, respectively), while there is no concordance (0%) between the phenotypic and genotypic profiles of *S.* Tennessee, Kimpese, and *S.* Cerro. Therefore, this indirectly indicates that the detection of antimicrobial resistance genes is related to multidrug resistance and, to a certain extent, with serovars. However, due to the small number of strains in certain serovars and the narrow sampling range, it is necessary to expand the sampling range to verify this conclusion.

PFGE typing was widely used in molecular epidemiological studies of a variety of pathogens worldwide, which can analyze bacterial chromosomal DNA directly with high discrimination and repeatability. Although the whole genome sequencing (WGS) is actually considered the gold standard method used to fingerprint the foodborne pathogens, PFGE is still considered among the major typing methods for the screening and discrimination of bacterial isolates, especially in the case of limited access to WGS. In fact, PFGE has been used by PulseNet for many years to track the source of diseases or food safety emergencies caused by bacterial infections, which has played an important role in the management of many public health incidents. In this study, a total of 118 *Salmonella* strains were analyzed and discriminated by PFGE-XbaI restriction. According to the obtained PFGE profiles, 22 different subtypes (PFGE profiles) were identified, which were grouped into 9 different cluster by taking a cut-off value of 90%. Additionally, the combination of the PFGE profiles and the serological results showed that different serovars shared the same PFGE profile (subtype) and that the isolates of each serovar presented more than one subtype divided into different clusters, which demonstrates that PFGE is unable to discriminate *Salmonella* serovars. These results are in agreement with those reported in previous studies [50,51,52]. Moreover, these findings suggest the presence of different sources of contamination, which are in line with previous studies [51,53]. In addition, our results showed that the differences in the PFGE patterns cannot be linked to the difference in antimicrobial-resistant genotypes among the same serovar. In fact, the targeted resistance genes are often carried by mobile genetic elements, including plasmids, integrons, and transposons, which cannot be discriminated by PFGE; moreover, in the case of antimicrobial resistance resulting from punctual mutations on bacterial DNA, the mutations are not at the restriction site of the XbaI enzyme and thus cannot be detected or discriminated by PFGE. This conclusion should be confirmed by further research.

## 4. Materials and Methods

### 4.1. Strains

This study was conducted with 120 strains selected from 504 *Salmonella* strains recovered during a previous study from 2139 chicken embryos from 28 hatcheries for breeding chickens in 9 cities of Henan province between August 2014 to April 2015 [8]. This study was focused on analyzing the non-Pullorum/non-Gallinarum *Salmonella* isolates (*n* = 120). However, the isolation, identification, and serotyping of *Salmonella* strains were performed as previously reported [8]. The studied *Salmonella* strains (*n* = 120) were divided into eleven serovars, including *S.* Enteritidis (*n* = 36; 30%), *S.* Blegdam (*n* = 18; 15%), *S.* Cerro (*n* = 18, 15%), *S.* Edinburg (*n* = 17; 14.17%), *S.* Thompson (*n* = 14; 11.67%), *S.* Tennessee (*n* = 9; 7.5%), *S.* Kimpese (*n* = 4; 3.33%), and one strain each (0.83%) for *S.* Entebbe, *S.* Tamilnadu, *S.* Fillmore, and *S.* Gatuni. Additionally, three other isolates belonging to *S.* Enteritidis (*n* = 2) and *S.* Blegdam (*n* = 1), which were isolated, identified, and stored by the Infectious disease Laboratory of Henan University of Animal Husbandry and Economics were also added to the PFGE analysis. *Escherichia* coli ATCC 25922 was donated by the Jiangsu Key Laboratory of Zoonoses, Yangzhou University. *Salmonella* Braenderup H9812 was provided by Henan CDC and was used as a standard strain for PFGE investigation.

### 4.2. Antimicrobial Susceptibility Test

The Kirby–Bauer (K-B) disc diffusion method was used to conduct the antimicrobial susceptibility tests, while the Clinical Laboratory Standards Institute (CLSI) [54] and veterinary recommendations of the Antibiogram Committee of the French Society of Microbiology (CA-SFM) [55] were used for the results interpretation of the studied isolates against 20 antimicrobial agents, representing 12 different classes: aminoglycosides (kanamycin: KAN, 30 μg; gentamicin: GEN, 10 μg; amikacin: AMK, 30 μg); Penicillins (ampicillin: AMP, 10 μg); β-lactams combination (amoxicillin/clavulanic acid: AMC, 20/10 μg); cephalosporins (ceftriaxone: CRO, 30 μg; ceftazidime: CAZ, 30 μg; cefazolin: CFZ, 30 μg); carbapenems (meropenem: MEM, 10 μg; imipenem: IPM, 10 μg); monobactams (aztreonam: ATM 30 μg); tetracyclines (tetracycline: TET 30 μg, oxytetracycline: OTC 30 μg); polypeptides (colistin: CST 50 μg); phenicols (chloramphenicol: CHL 30 μg); quinolones (enrofloxacin: ENR, 5 μg; ciprofloxacin: CIP, 5 μg); sulphonamides (trimethoprim/sulfamethoxazole: SXT, 1.25/23.75 μg; sulfisoxazole: SIZ, 250 μg); and nitrofurans (nitrofurantoin: NIT, 300 μg). However, the results obtained by the disc diffusion method against colistin were confirmed by means of the broth dilution method, according to the recommendations of CA-SFM (Supplementary Figure S1) [55]. To facilitate analysis, isolates showing intermediate results were classified as resistant strains. Moreover, the phenotypic evaluation of the extended-spectrum β-lactamases- (ESBLs) producing strains was performed by double-disk synergy test according to the recommendation of the Clinical and Laboratory Standards Institute [54].

### 4.3. Detection of Antimicrobial Resistance Genes

In this study, the genomic DNA was extracted using FastPure Bacteria DNA Isolation Mini Kit (Vazyme Biotech Co.,Ltd) according to the user manual. The amplification of 37 genes encoding resistance to different antimicrobial categories was conducted by 2×Easy KOD PCR SuperMix (Zhejiang Easy-Do Biotech Co., Ltd) subsequently. Among them, twelve genes encoding ESBLs (*bla*_TEM_, *bla*_SHV_, *bla*_CTX-M-1_, *bla*_CTX-M-2_, *bla*_CTX-M-3_, *bla*_CTX-M-8_, *bla*_CTX-M-9_, *bla*_CTX-M-25_, *bla*_OXA-1_, *bla*_OXA-2_, *bla*_OXA-10,_ and *bla*_PSE_), with ten encoding resistance to aminoglycosides (*armA*, *rmtA*, *rmtB*, *rmtC*, *rmtD*, *rmtE*, *npmA*, *aac(3)-IV*, *aac6′-Ib,* and *aadA1-like*), six encoding resistance to cephalosporins (*bla*_MOX_, *bla*_CIT_, *bla*_DHA_, *bla*_ACC_, *bla*_EBC,_ and *bla*_FOX_), three encoding resistance to sulphonamides (*sul1*, *sul2,* and *sul3*), and six encoding resistance to tetracyclines (*tetA*, *tetG*, *tetX*, *tetB*, *tetC,* and *tetR*). Primer design and PCR amplification conditions were summarized in Table S1. A part of the amplified products was randomly selected and sequenced by Sangon Biotech after PCR amplification (Sangon Biotech (Shanghai) Co., Ltd). Nucleotide sequence comparisons were performed using the BLAST software (NCBI, Bethesda, MD, USA) available from the NCBI website (https://blast.ncbi.nlm.nih.gov/Blast.cgi, accessed on 18 September 2021), DNAStar software (DNASTAR, Inc., Madison, WI, USA) as well as MEGA 5.1 software (Pennsylvania State University, State College, PA, USA).

### 4.4. Pulsed-Field GEL Electrophoresis Analysis

Pulsed-field gel electrophoresis (PFGE) was conducted according to the standardized protocol for *Salmonella* PFGE from PulseNet USA, a surveillance network of public health laboratories across the United States and other countries. Specifically, bacterial suspensions of *Salmonella* isolates were prepared from overnight cultures, fixed in agarose plugs, and lysed to liberate the DNA in agarose plugs. Then, DNA was digested with 50U XbaI restriction enzyme in a 37 °C water bath for at least 2 h. Afterward, the restricted fragments were separated in 1% agarose using Chef Mapper pulse-field gel electrophoresis, according to the following conditions: the buffer solution was 0.5× TBE or Tris/Borate/EDTA, the electrophoresis time was 18 h, the electrophoresis temperature was 14 °C, and the pulse time was 2.16 s to 63.8 s. After migration, the gel was stained with ethidium bromide and visualized under ultraviolet (UV) light, and an image was taken with a digital camera and was stored for further analysis.

The obtained PFGE profiles were analyzed by BioNumerics 7.6 software and the Dice coefficient, and the dendrogram was generated by the unweighted pair-group method with arithmetic means (UPGMA) based on the 1.5% position optimization and tolerance values. The band of 100% similarity was regarded as the same PFGE type.

### 4.5. Statistical Analysis

Two-way ordinary ANOVA was used to test the significant difference of antimicrobial resistance and ESBLs being produced among the serovars. *p* values less than 0.05 were considered statistically significant. GraphPad Prism 7 software (San Diego, CA, USA) was used for data analysis and the generation of the figures.

## 5. Conclusions

This study investigated the antimicrobial resistance profiles, epidemiological characteristics of resistance genes, and PFGE-XbaI typing of *Salmonella* isolates from chicken embryos in Henan province, China. Our findings showed that the studied *Salmonella* isolates presented high resistance to antimicrobial agents with diversified multidrug-resistant patterns, which requires more attention to the dissemination of multidrug-resistant strains, especially those producing ESBLs. Moreover, we demonstrated the coherence between genotypic and phenotypic resistance of the studied strains, while the PFGE patterns analysis provided 22 different subtypes grouped into 9 clusters with high diversity in each serovar, suggesting multiple sources of contamination. Therefore, we recommend the implementation of systematic and judicious medication management in the breeding industry to mitigate the development of drug-resistant and multidrug-resistant strains.

## Data Availability

The data presented in this study are available in Supplementary Material.

## Supplementary Materials

The following are available online at https://www.mdpi.com/article/10.3390/antibiotics10101156/s1, Figure S1: The distribution of minimum inhibitory concentration (MIC) values among the examined Salmonella isolates (*n* = 120) against Colistin (CST). The dashed line indicates the cutoff level of the MIC, in which the lowest values correspond to susceptibility and the highest values correspond to the resistance, Table S1: Primers used for the detection of antimicrobial resistance genes.
