# Supplementary material for: Antimicrobial Resistance Profiles and Genetic Typing of Salmonella Serovars from Chicken Embryos in China
Source: Antibiotics (Basel). 2021 Sep 24;10(10):1156. doi: 10.3390/antibiotics10101156 (PMC8532839; doi:10.3390/antibiotics10101156)
Supplement: Supplementary file 1 [file antibiotics-10-01156-s001.zip › antibiotics-1361843-SI.pdf]

Antimicrobial resistance profiles and genetic typing of *Salmonella* serovars from chicken embryos in China

Yaohui Xu [1,†], Xiao Zhou [2,†], Zenghai Jiang [1], Yaru Qi [1], Abdelaziz Ed-Dra [3], Min Yue [2,3,4,5,*]

[1] College of Veterinary Medicine, Henan University of Animal Husbandry and Economy, Zhengzhou 450046, China; 80588@hnuahe.edu.cn (Y.X.); 80147@hnuahe.edu.cn (Z.J.); q15837181937@163.com (Y.Q.)
[2] Department of Veterinary Medicine & Institute of Preventive Veterinary Sciences, Zhejiang University College of Animal Sciences, Hangzhou 310058, China; xiaozhou@zju.edu.cn
[3] Hainan Institute of Zhejiang University, Sanya 572025, China; abdelaziz_iaa@yahoo.fr
[4] National Clinical Research Center for Infectious Diseases, National Medical Center for Infectious Diseases, State Key Laboratory for Diagnosis and Treatment of Infectious Diseases, The First Affiliated Hospital, College of Medicine, Zhejiang University, Hangzhou 310003, China
[5] Zhejiang Provincial Key Laboratory of Preventive Veterinary Medicine, Hangzhou 310058, China
* Correspondence: myue@zju.edu.cn
† These authors contributed equally to this work.

Supplementary Figures

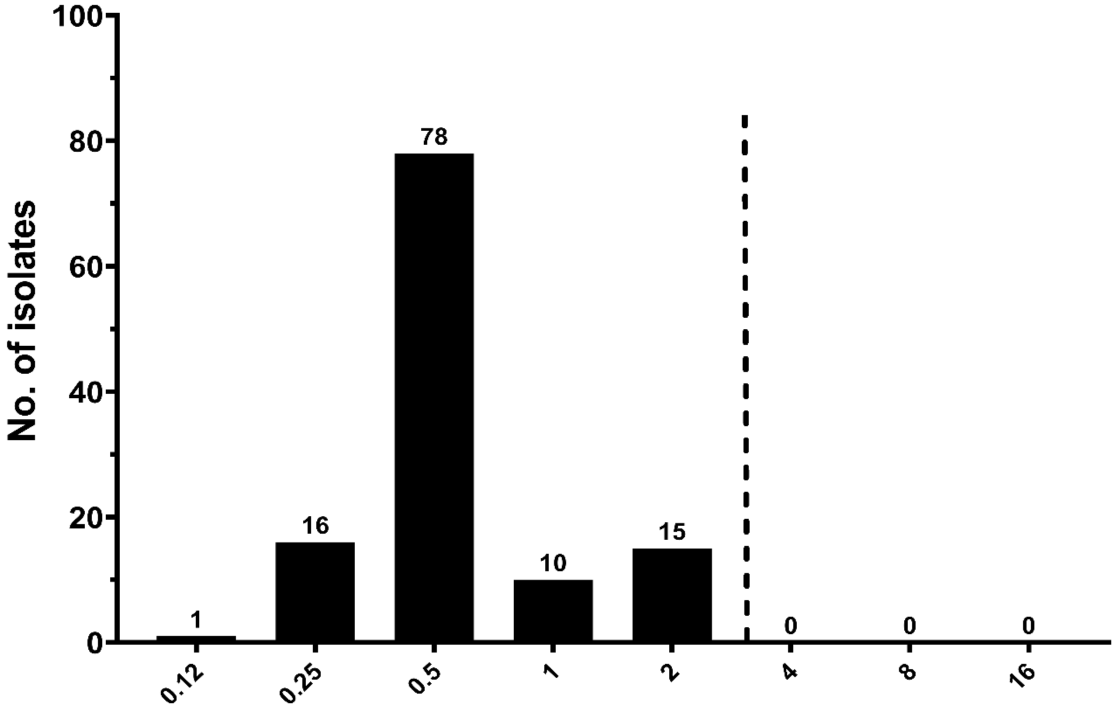

Figure S1. The distribution of minimum inhibitory concentration (MIC) values among the examined *Salmonella* isolates (n=120) against Colistin (CST). The dashed line indicates the cutoff level of the MIC, in which the lowest values correspond to susceptibility and the highest values correspond to the resistance.

Supplementary Tables

Table S1: Primers used for the detection of antimicrobial resistance genes.

Target	Forward primer (5′–3′)	Reverse primer (5′–3′)	Annealing Temperature (°C)	Product size (bp)	References
β-Lactamases and ESBLs:					
bla$_{\text{TEM}}$	GTATTCAACATTTCCGTGTCG	CCAATGCTTAATCAGTGAGGC	55	854	[1]
bla$_{\text{SHV}}$	CCCTGTTAGCCACCCTGCCG	CGTTGCCAGTGCTCGATCAGC	68	829	[1]
bla$_{\text{CTX-M-1}}$	GCTGTTGTTAGGAAGTGTGCCGC	GCCGCCGACGCTAATACATC	63	798	[1]
bla$_{\text{CTX-M-2}}$	AAATGTGCTGCTCCTTTCGTGAGC	AGGGTTCGTTGCAAGACAAGACTG	55	1122	[2]
bla$_{\text{CTX-M-3}}$	GTTACAATGTGTGAGAAGCAG	CCGTTTCCGCTATTACAAAC	55	1018	[3]
bla$_{\text{CTX-M-8}}$	TAACGCACAGACGCTCTACC	TGGCTGGGTGAAGTAAGTGAC	55	637	[4]
bla$_{\text{CTX-M-9}}$	GATCAGTCAGTGGGATAGTTT	TACTCGGCGTTAACTGATTA	47	671	[5]
bla$_{\text{CTX-M-25}}$	GCGATGTTAATGACGACAGC	AACCGTCGGTGACAATTCTG	55	847	[4]
bla$_{\text{OXA-1}}$	ACACAATACATATCAACTTCGC	AGTGTGTTTAGAATGGTGATC	55	814	[3]
bla$_{\text{OXA-2}}$	TTCAAGCCAAAGGCACGATAG	TCCGAGTTGACTGCCGGGTTG	60	703	[3]
bla$_{\text{OXA-10}}$	CGTGCTTTGTAAAAGTAGCAG	CATGATTTTGGTGGGAATGG	55	389	[3]
bla$_{\text{PSE}}$	TGCTTCGCAACTATGACTAC	AGCCTGTGTTTGAGCTAGAT	55	438	[3]
Cephalosporins:					
bla$_{\text{MOX}}$	GCTGCTCAAGGAGCACAGGAT	CACATTGACATAGGTGTGGTGC	55	520	[6]
bla$_{\text{CIT}}$	TGGCCAGAACTGACAGGCAAA	TTTCTCCTGAACGTGGCTGGC	55	462	[6]
bla$_{\text{DHA}}$	AACTTTCACAGGTGTGCTGGGT	CCGTACGCATACTGGCTTTGC	55	405	[6]
bla$_{\text{ACC}}$	AACAGCCTCAGCAGCCGGTTA	TTCGCCGCAATCATCCCTAGC	55	346	[6]
bla$_{\text{EBC}}$	TCGGTAAAGCCGATGTTGCGG	CTTCCACTGCGGCTGCCAGTT	55	302	[6]
bla$_{\text{FOX}}$	AACATGGGGTATCAGGGAGATG	CAAAGCGCGTAACCGGATTGG	55	190	[6]
Aminoglycosides:					
armA	CAAATGGATAAGAATGATGTT	TTATTTCTGAAATCCACT	55	777	[7]
rmtA	ATGAGCTTTGACGATGCCCTA	TCACTTATTCCTTTTTATCATG	55	756	[7]
rmtB	ATGAACATCAACGATGCCCT	CCTTCTGATTGGCTTATCCA	55	769	[7]
rmtC	CGAAGAAGTAACAGCCAAAG	ATCCCAACATCTCTCCCACT	55	711	[7]
rmtD	CGGCACGCGATTGGGAAGC	CGGAAACGATGCGACGAT	55	401	[7]
rmtE	ATGAATATTGATGAAATGGTTGC	TGATTGATTTCCTCCGTTTTTG	55	819	[7]
npmA	CTCAAAGGAACAAAGACGG	GAAACATGGCCAGAAACTC	55	641	[8]
aac(3)-IV	GTTACACCGGACCTTGGA	AACGGCATTGAGCGTCAG	55	674	[9]

aac6′-Ib	TTGCGATGCTCTATGAGTGGCTA	CTCGAATGCCTGGCGTGTTT	55	482	[10]
aadA1-like	GTGGATGGCGGCCTGAAGCC	ATTGCCCAGTCGGCAGCG	55	527/1916	[9]
Sulphonamides:					
sul1	TGGCGTCGCGACTGCGAAAT	TGGTGACGGTGTTCGGCATTCT	55	813	[11]
sul2	GTTTCTCCGATGGAGGCCGGT	AGCGAGGTTTCGGGAGCAGC	55	517	[11]
sul3	GATAGTTTTTCCGATGGAGG	GAAGCCCATACCCGGATCAA	55	495	[11]
Tetracyclines:					
tetA	TGGTCCGGAGGCCAGACGTG	TTCCGAGCATGAGTGCCCGC	55	867	[11]
tetG	TCTTGCAGGAGCCGCAGTCGAT	GGCCGGCATGCCAACACCC	55	721	[11]
tetX	CAATAATTGGTGGTGGACCC	TTCTTACCTTGGACATCCCG	55	468	[11]
tetB	AAATAACAGCAAACAGTAATGG	AAGTAGGGGTTGAGACGCAGCTA	55	493	[12]
tetC	TCTAACAATGCGCTCATCGT	GGTTGAAGGCTCTCAAGGGC	55	589	[12]
tetR	AGGACGACGGTGCTTGCT	ATGAGGACTGGCGGGTGT	55	295	[12]

References

1. González-Sanz, R.; Herrera-León, S.; de la Fuente, M.; Arroyo, M.; Echeita, M.A. Emergence of extended-spectrum β-lactamases and AmpC-type β-lactamases in human Salmonella isolated in Spain from 2001 to 2005. *J. Antimicrob. Chemother.* **2009**, *64*, 1181–1186, doi:10.1093/jac/dkp361.
2. Garcia, D.D.O.; Doi, Y.; Szabo, D.; Adams-Haduch, J.M.; Vaz, T.M.I.; Leite, D.; Padoveze, M.C.; Freire, M.P.; Silveira, F.P.; Paterson, D.L. Multiclonal outbreak of Klebsiella pneumoniae producing extended-spectrum β-lactamase CTX-M-2 and novel variant CTX-M-59 in a neonatal intensive care unit in Brazil. *Antimicrob. Agents Chemother.* **2008**, *52*, 1790–1793, doi:10.1128/AAC.01440-07.
3. Riaño, I.; Moreno, M.A.; Teshager, T.; Sáenz, Y.; Domínguez, L.; Torres, C. Detection and characterization of extended-spectrum β-lactamases in Salmonella enterica strains of healthy food animals in Spain. *J. Antimicrob. Chemother.* **2006**, *58*, 844–847, doi:10.1093/jac/dkl337.
4. Eller, C.; Simon, S.; Miller, T.; Frick, J.S.; Prager, R.; Rabsch, W.; Guerra, B.; Werner, G.; Pfeifer, Y. Presence of β-lactamases in extended-spectrum-cephalosporinresistant salmonella enterica of 30 different serovars in germany 2005-11. *J. Antimicrob. Chemother.* **2013**, *68*, 1978–1981, doi:10.1093/jac/dkt163.
5. Coque, T.M.; Oliver, A.; Pérez-Díaz, J.C.; Baquero, F.; Cantón, R. Genes encoding TEM-4, SHV-2, and CTX-M-10 extended-spectrum β-lactamases are carried by multiple Klebsiella pneumoniae clones in a single hospital (Madrid, 1989 to 2000). *Antimicrob. Agents Chemother.* **2002**, *46*, 500–510, doi:10.1128/AAC.46.2.500-510.2002.
6. Pérez-Pérez, F.J.; Hanson, N.D. Detection of plasmid-mediated AmpC β-lactamase genes in clinical isolates by using multiplex PCR. *J. Clin. Microbiol.* **2002**, *40*, 2153–2162, doi:10.1128/JCM.40.6.2153-2162.2002.
7. Granier, S.A.; Hidalgo, L.; Millan, A.S.; Escudero, J.A.; Gutierrez, B.; Brisabois, A.; Gonzalez-Zorn, B. ArmA methyltransferase in a monophasic Salmonella enterica isolate from food. *Antimicrob. Agents Chemother.* **2011**, *55*, 5262–5266, doi:10.1128/AAC.00308-11.
8. Fritsche, T.R.; Castanheira, M.; Miller, G.H.; Jones, R.N.; Armstrong, E.S. Detection of methyltransferases conferring high-level resistance to aminoglycosides in enterobacteriaceae from Europe, North America, and Latin America. *Antimicrob. Agents Chemother.* **2008**, *52*, 1843–1845, doi:10.1128/AAC.01477-07.
9. Guerra, B.; Junker, E.; Miko, A.; Helmuth, R.; Mendoza, M.C. Characterization and localization of drug resistance determinants in multidrug-resistant, integron-carrying Salmonella enterica serotype typhimurium strains. *Microb. Drug Resist.* **2004**, *10*, 83–91, doi:10.1089/1076629041310136.
10. de Toro, M.; Rojo-Bezares, B.; Vinué, L.; Undabeitia, E.; Torres, C.; Sáenz, Y. In vivo selection of aac(6′)-Ib-cr and mutations in the gyrA gene in a clinical qnrS1-positive Salmonella enterica serovar Typhimurium DT104B strain recovered after fluoroquinolone treatment. *J. Antimicrob. Chemother.* **2010**, *65*, 1945–1949, doi:10.1093/jac/dkq262.
11. Lu, S.; Juan, W.; XiuMei, H.; ZhiNa, Q.; JunWei, W.; RuiMei, Y.; ZengRen, Z. Detection of Salmonella resistance genes and correlation analysis of drug resistance in broiler slaughter production chain. *China Anim. Heal. Insp.* **2017**, *34*, 35–42.
12. YaoHui, X.; TongWei, D.; YaRu, Q.; WenLong, H.; JianZhou, L.; ZengHai, J. Isolation, identification and antimicrobial resistance of Salmonella from dead chicken embryos in Sanhuang breeder farms. *Acta Vet. Zootech. Sin.* **2018**, *49*, 1081–1088.
